# Extensive carotid atherosclerosis and the diagnostic accuracy of coronary risk calculators

**DOI:** 10.1016/j.pmedr.2017.03.006

**Published:** 2017-03-14

**Authors:** Michel Romanens, Martin Bødtker Mortensen, Isabella Sudano, Thomas Szucs, Ansgar Adams

**Affiliations:** a Vascular Risk Foundation, Olten, Switzerland; b Department of Cardiology, Aarhus Universitetshospital, Aarhus, Denmark; c University Heart Center, Cardiology, University Hospital, Zurich, Switzerland; d European Centre of Pharmaceutical Medicine (ECPM), Basel, Switzerland; e BAD Gesundheitsvorsorge und Sicherheitstechnik GmbH, Bonn, Germany

**Keywords:** Atherosclerosis, Risk-prediction, Statin-indication, Carotid plaque

## Abstract

Preventive therapy in primary care is guided by risk thresholds for future cardiovascular events. We aimed to assess whether the sensitivity of various risk calculators for the detection of subclinical carotid atherosclerosis (TPA80) could be improved by lowering risk thresholds in younger age groups. We compared sensitivity, specificity, and discriminatory performance of SCORE, SCORE-HDL, PROCAM, AGLA, FRAM and PCE coronary risk calculators to detect total plaque area > 80 mm2 (TPA80), a coronary risk equivalent, in age groups 40–55, 56–65, 66–75 from Germany (DE, *N* = 2942) and Switzerland (CH, *N* = 2202) during the years 2002 to 2016. All calculators showed good to moderate discriminatory performance to detect TPA80 with AUC ranging from 0.74 (CH-AGLA) to 0.87 (DE- SCORE), but the sensitivity of high risk risk thresholds varied widely from 39% for DE-FRAM-CVD to 5% for CH-AGLA. Lowering of the risk threshold increased sensitivity substantially at the expense of minor losses in specificity, but the sensitivity generally remained < 45% at the 90% specificity threshold.

Current risk thresholds of American and European coronary risk calculators have a low sensitivity to detect TPA80 in younger individuals.

## Highlights

• Low sensitivity of European and American risk calculators in two independent populations
• Relatively high prevalence of advanced carotid atherosclerosis in people aged 40–65
• New thresholds for intermediate or high risk in younger subjects

## 1 Introduction

Tests used in clinical and preventive medicine have a certain sensitivity (disease detection rate in those with disease) and specificity (rate of exclusion of a disease in those without the disease). In preventive medicine, 10-year risk estimates are calculated and in general lower treatment thresholds are associated with a higher sensitivity and a lower specificity. In the Framingham Offspring Study coronary risk prediction was improved by reducing risk thresholds in younger subjects (Navar-Boggan et al., 2015a).

While a clinician's preventive efficacy is dependent on meaningful sensitivity thresholds, tests need proof with regards to their discriminatory value. By taking the whole range of test results, a plot of sensitivity and specificity is created by receiver operating curves (ROC) to detect those with a future event. Acceptable area under the curve (AUC) is usually larger than 0.80.

Such calculations are based on cardiovascular events occurring over time. By definition, such an approach translates observations from the past into the present. A “present time” validation to assesses the accuracy of coronary risk calculators can be derived from patients admitted for a first myocardial infarction, where a very low sensitivity was revealed for the European calculator (SCORE-CVD (Conroy et al., 2003)) risk threshold of 5% (Mortensen et al., 2015, Mortensen and Falk, 2014). Instead of waiting until a myocardial infarction occurs, atherosclerosis imaging also offers a “present time” validation for coronary risk calculators by measuring the total carotid plaque burden. Such information can therefore be used to test risk calculators for their performance before the occurrence of an acute coronary event and may help to define sensitivity cutoffs in those populations, where atherosclerosis burden information is available (Arbab-Zadeh & Fuster, 2015).

For the purpose of this study, we used a total plaque area of greater than or equal to 80 mm^2^ (TPA80), for which a coronary risk of > 20% was found in a long-term observational study (median observation time 15.4 years) in 6257 subjects from the Norwegian Tromsø area (Hald et al., 2013) in order to test the performance of various risk calculators for their sensitivity and specificity in three different age.

## 2 Materials and methods

### 2.1 Subject selection

Subjects were assessed at the practice based level as described elsewhere (Romanens et al., 2014, Romanens et al., 2011). In the Swiss (CH) Imaging Center in Olten, subjects were referred by their primary care physician (57%) or self-referred to the vascular risk foundation (43%; www.varifo.ch). In the German (DE) Center in Koblenz, all subjects were referred within a workplace medicine setting (Adams & Bojara, 2015). Subjects had to be free of cardiovascular symptoms or diseases. The medical history was assessed, laboratory values, blood pressure determined locally and entered into a data spread-sheet (Excel, Microsoft, Richmond, USA).

### 2.2 Ethical aspects

Subjects with self-referral to the Vascular Risk Foundation gave written consent. The study protocol was approved by the local ethical committee of Solothurn, Switzerland. Practice based subjects were entered into an anonymized study registry, for which current legislation in Switzerland and Germany does not require formal ethical committee consent.

### 2.3 Carotid imaging

Burden of longitudinal carotid plaque surface was imaged with a high resolution ultrasound linear transducer probe (7.5–12.0 MHz), which identified plaques with intimal thickening ≥ 1.0 mm. The longitudinal area of all plaques was summed up to the total plaque area (TPA) in mm^2^. All TPA measurements were made by A.A. in Koblenz and by M.R. in Olten. A TPA ≥ 80 mm^2^ (TPA80) defined a coronary risk equivalent (risk > 20% for fatal and non-fatal myocardial infarction in 10 years) (Hald et al., 2013). Intraobserver reproducibility (MR) was tested for the right carotid artery in 57 patients with a correlation coefficient of r^2^ 0.964 (left carotid artery: r^2^ 0.944, both arteries r^2^ 0.986). For the cutoffs of TPA 0–9 mm^2^, 10–49 mm^2^, 50–99 mm^2^ and > 100 mm^2^ Kappa value was 0.69 (0.54–0.84 95% CI).

### 2.4 Computation of risk

Cardiovascular risk was computed using the published risk formulae in an Excel spread sheet. We used the European Society of Cardiology risk calculators for low risk populations (SCORE and SCORE-HDL (Descamps et al., 2012)), the pooled cohort equation (PCE (Robinson & Stone, 2015)) and the Framingham risk calculator for major cardiac (FRAM-CHD (D'Agostino et al., 2008)) and major cardiovascular events (FRAM-CVD (D'Agostino et al., 2008)). The German PROCAM risk (Assmann et al., 2007) was calculated manually online, since the algorithm is not published. For Switzerland, PROCAM risk was multiplied by the factor 0.7 (CH-AGLA, according to the Swiss AGLA guidelines 2014 (Eckardstein, 2014)). SCORE risk was calculated using the algorithm published by Conroy (Conroy et al., 2003) and the SCORE-HDL (Cooney et al., 2009) risks were calculated as previously described by Descamps (Descamps et al., 2012).

### 2.5 Statistics

We used MedCalc software (Version 13.3.3.0) to calculate ROC curves and their comparisons (MedCalc Software bvba, 2013). For comparison of risk calculators, equivalent SCORE risk was set to be four times lower than in the remainder, therefore, a PROCAM or FRAM risk of 20% would correspond to an SCORE risk of 5%. Level of statistical significance was set at *p* < 0.05.

## 3 Results

### 3.1 Patient characteristics

We assessed 2202 healthy Swiss and 2942 healthy German subjects. The characteristics of the study subjects are shown in Table 1. The Swiss group was older than the German group (57 ± 9 versus 46 ± 10 years) with more women (49% versus 34%). Average 10-year risk among groups was low. Prevalence of TPA80 was 22% in Switzerland and 15% in Germany. Lipid profiles were comparable.

### 3.2 Prevalence of TPA80

The prevalence of TPA80 was low in Swiss women aged 40–55 years (4%), but increased to 14% and 36% in the two remaining age groups. For men, TPA80 was prevalent in all age groups above the 15% level, and was present in 57% in Swiss men aged 66 to 75 years (Table 2).

### 3.3 Sensitivity and Specificity of high risk coronary risk thresholds for the detection of TPA80

Using high risk thresholds for high coronary risk (5% for the SCORE and SCORE-HDL risk calculators, 20% for the remaining cardiovascular risk calculators), global sensitivity to detect TPA80 showed some variability, but was generally below 20% in Switzerland and Germany. Of note, CH-AGLA had a sensitivity of only 5% (Table 3).

### 3.4 C-Statistics of coronary risk calculators (Fig. 1)

We found that the performance of all cardiovascular risk calculators was similar in Switzerland and Germany, but with slightly higher values for Germany and with significant differences among calculators (Supplemental Table I): especially CH-AGLA showed a significantly lower area under the curve (AUC 0.743), while the same was true for the DE-PCE risk calculator (AUC 0.769). Good performance was found both for the FRAM-CVD, SCORE and SCORE-HDL risk calculators.

### 3.5 Effect of different risk thresholds on sensitivity and specificity to detect TPA80 by sex and age groups for SCORE, PROCAM and CH-AGLA

Supplemental Tables II to IV show the sensitivity and specificity by age groups and various risk thresholds for PROCAM and SCORE for women and men respectively to detect TPA80. Supplemental Tables V and VI sensitivity of various risk thresholds among different risk algorithms. By increasing risk thresholds sensitivity is reduced to zero or near zero, with specificities at near 100% or 100%.

## 4 Discussion

We assessed sensitivity, specificity and discriminatory performance (area under curve, AUC) as well as predictive values of several American and European risk calculators to detect a coronary risk equivalent defined by the total carotid plaque burden (TPA80) in a practice based setting of 5144 subjects from Koblenz (*N* = 2942) and Olten region (*N* = 2202). The prevalence range of TPA80 was between 4% in younger women and 57% in elderly men (Table 2).

**Fig. 1:**
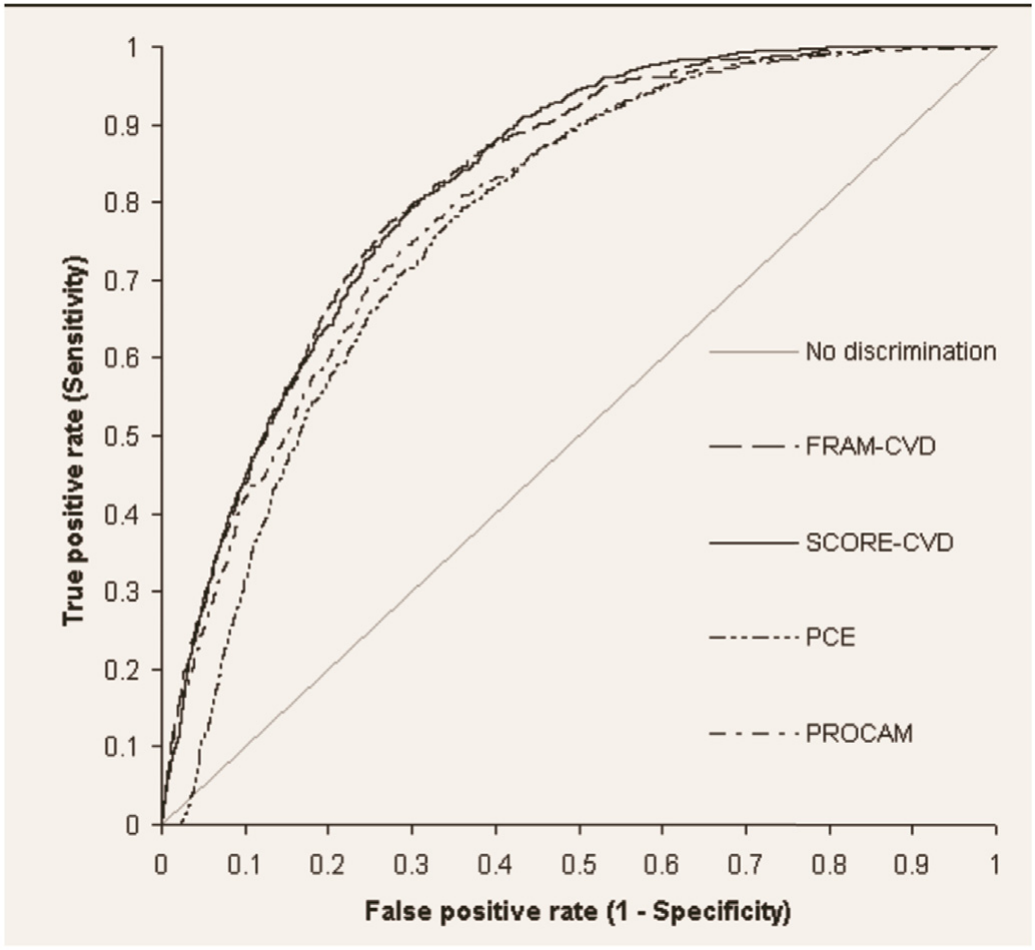
Summarized discriminatory performance of FRAM-CVD, SCORE-CVD, PCE and PROCAM coronary risk calculators to detect total plaque area > 80 mm^2^ (TPA80) in 5′144 primary prevention subjects.

Results from ROC curves (Fig. 1) showed acceptable discriminatory performance to detect TPA80 with (0.74 for CH-AGLA to 0.87 for DE-SCORE-HDL, Supplemental Table I). Although ROC analysis show generally good discriminatory performance of coronary risk calculators externally (DeFilippis et al., 2015), reliance on recalibration based on predicted-to-observed (P/O) event ratio in cohorts where true negatives by far outweigh numerically true positives usually creates a calibration in favor of true negatives (Navar-Boggan et al., 2015a, Mortensen et al., 2015, DeFilippis et al., 2015). Such a down-calibration was performed with the Swiss AGLA coronary risk calculator, thus reducing coronary risk as compared to Germany by 30%.

The high-risk threshold for SCORE (5%) and for the remainder of the calculators (20%) had sensitivities below 30% except for CH-FRAM-CVD (39%) and DE-FRAM-CVD (39%). Our results indicate that subjects with a coronary risk equivalent in mid-life remain frequently undetected (sensitivity between 5% and 39%, Table 3). Therefore, the conundrum of risk prediction is not resolved (McEvoy et al., 2014). Risk thresholds should maintain a specificity of at least 90% and Supplemental Tables II - IV show that sensitivities usually remain below 25% (especially in younger women). Our data could help future guideline committees to use lower decision thresholds in order to detect higher risk individuals with an increased sensitivity.

Use of total plaque burden is accurate to predict cardiovascular risk (Baber et al., 2014, Spence and Hackam, 2010, Gottesman et al., 2014). As confirmed by in the long-term Tromsø study, TPA80 – a rapid and cheap test that does not require expensive radiology, radiation exposure or software – is a high risk finding for incident myocardial infarction (Hald et al., 2013). TPA of 40 ± 22 mm^2^ derived from the right carotid artery was associated with an unadjusted coronary risk of 23.9% (95%CI: 21.2–27.1) in 10 years. The Hazard Ratio per 1-SD increase in TPA (2.43 mm^2^) was 1.23 (95%CI: 1.15–1.32) using age as time scale and adjustments for sex, body mass index, smoking, total cholesterol, high-density lipoprotein cholesterol, diabetes mellitus, and hypertension.

Earlier preventive therapy may better protect against harm due to atherosclerosis later in life, which has been shown for both arterial hypertension (Gottesman et al., 2014) and hypercholesterolemia (Navar-Boggan et al., 2015b). Statin treatment is still highly effective even in the fittest (Kokkinos et al., 2012). A five year treatment of 1000 healthy men aged between 45 and 54 years with pravastatin (40 mg/day) saved the British Health Care System £710,000 over a 15-year period and savings were even higher (£840′000) in those at low risk (7.5% in 10 years risk) (Mc Connachie et al., 2014). A 50-year old woman with a CH-FRAM-CVD risk of 7% and TPA80 has an arterial age of 75 years ^7^ and a posttest risk of 35% (Romanens M. VARIFO Cardiovascular Risk Calculator. 2016. Available from: www.docfind.ch/GPRisk.xlsx).

A low sensitivity for clinical events were confirmed by the Copenhagen General Population Study, where 68 fatal and 767 fatal and non-fatal cardiovascular events occurred over an observation time of 7 years. Sensitivity at the 5% SCORE level was 42% and 26% respectively (Mortensen et al., 2015).

We were able to replicate the results of the Framingham Offspring Study for the coronary risk calculators PROCAM/AGLA and SCORE (Navar-Boggan et al., 2015a). Only at a risk threshold of 0.75% did the SCORE calculator have acceptable sensitivities and specificities in men and women aged 40–55. We confirm the study results by Mortensen et al., who found similar results: of 162 men and 85 women with a first myocardial infarction, only 8% and 1% respectively would have qualified for a statin treatment before the event when using a cutoff of SCORE ≥ 5% (Mortensen & Falk, 2014).

For SCORE and SCORE-HDL, we were able to replicate the results from the Copenhagen General Population Study, where lower risk thresholds of 1% rather than of 5% 10 year risk increased the sensitivity for fatal and non-fatal cardiovascular events from SCORE 26% (SCORE-HDL 17%) to 79% (SCORE-HDL 71%) in men and women aged 40–65 years (Mortensen et al., 2015). Counter-intuitively we also confirmed the finding that the addition of HDL in the SCORE model reduced the sensitivity of SCORE-HDL when compared to SCORE (Supplemental Tables V and VI). In a recent survey involving 44,889 subjects aged 40–75 years a SCORE sensitivity threshold of 2.4% was best suited for assigning a preventive therapy with statins (Mortensen et al., 2017).

### 4.1 Study limitations

Our study examines practice based groups of subjects, which cannot be extrapolated to the entire population. This may also be viewed as a potential strength of the study, since practice based subjects may serve as an external validation for coronary risk calculators.

The images were obtained within a clinical setting as part of routine measurements by two different observers. However, the congruence as outlined in Table 2 of the findings from Koblenz and Olten may be viewed as a mutual validation. The total plaque area, a measure of the total carotid plaque burden anticipated the results of the IMPROVE-IT study, thus confirming the high prognostic validity of such measurements (Cannon et al., 2015, Spence, 2008, Spence, 2012, Bogiatzi and Spence, 2012).

We do not present hard coronary outcome data in this study, however, there is high confidence for TPA80 being a true coronary risk finding and we were able to confirm the poor sensitivity performance of SCORE-HDL versus SCORE, originally described in the Copenhagen General Population Study (Mortensen et al., 2015). Our approach is in line with the recommendations of American and European guidelines, to treat atherosclerosis reported by medical imaging (Mansia et al., 2007, De Backer et al., 2003, Stone et al., 2014). Validation by plaque imaging as a tool to test the 2013 ACC/AHA guideline on the treatment of blood cholesterol to reduce atherosclerotic cardiovascular disease risk guidelines is widely accepted for the PCE calculator (Robinson and Stone, 2015, Johnson and Dowe, 2014). Empirical evidence has shown that the regression of total plaque area over time due to medical intervention is associated with a statistically significant reduction of cardiovascular events (Spence et al., 2002).

## 5 Conclusions

In our practice based group of 5144 subjects without cardiovascular disease, we find an acceptable discriminatory performance (ROC analysis) of all coronary risk calculators to detect TPA80. In coronary risk prevention at the individual level, where high sensitivity should exist to detect subjects with a coronary risk equivalent derived from the total carotid plaque burden, we observed a poor sensitivity of risk factor-based assessments when using recommended risk thresholds especially in subject aged 40–65. We find that improvement in sensitivity can be achieved by lowering risk thresholds without notable losses in specificity.

## Sources of Funding, conflict of interest & disclosures

None declared.

## Figures and Tables

**Table 1:** Baseline Characteristics, average and prevalence of cardiovascular risk and average TPA for Switzerland (CH) and Germany (DE).

Country	CH			DE		
Number of subjects (N)	2202			2942		
Female, N, %	1082		49%	989		34%
Mean age (N ± SD)	57	±	9	46	±	10
Family history for CAD (N, %)	386		18%	660		22%
Current smoker (N, %)	458		21%	770		26%
Blood pressure systolic, mm Hg mean ± SD	129	±	16	123	±	16
TPA mm^2^ mean ± SD	52	±	50	36	±	50
Individuals with TPA ≥ 80 mm^2^ (N, %)	484		22%	452		15%
Total cholesterol, mmol/l, mean ± SD	5.9	±	1.2	5.9	±	1.2
HDL cholesterol, mmol/l, mean ± SD	1.5	±	0.5	1.4	±	0.4
LDL cholesterol, mmol/l, mean ± SD	3.7	±	1.0	3.8	±	0.9
Triglycerides, mmol/l, mean ± SD	1.5	±	0.9	1.7	±	1.2
FRAM-CHD	9.0	±	7.1	6.5	±	6.0
% individuals with risk < 10%	67%			79%		
FRAM-CVD	13.2	±	9.8	9.3	±	8.4
% individuals with risk < 10%	47%			66%		
SCORE	2.4	±	2.6	1.1	±	1.4
% individuals with risk < 5%	87%			99%		
SCORE-HDL	1.8	±	2.0	0.8	±	1.2
% individuals with risk < 5%	93%			99%		
PCE	8.0	±	7.4	7.8	±	13.8
% individuals with risk < 10%	70%			80%		
PROCAM	6.2	±	7.3	4.3	±	6.2
% individuals with risk < 10%	81%			87%		
AGLA	4.3	±	5.1			
% individuals with risk < 10%	89%					

**Table 2:** Prevalence (N, %) of TPA80 by age groups and sex for Switzerland (CH) and Germany (DE).

		CH		DE	
TPA80	Age group	**N**	**%**	**N**	**%**
Women	40–55	18	4.5	27	4.4
	56–65	60	14.0	45	25.7
	66–75	78	36.3		
					
Men	40–55	79	15.7	195	17.5
	56–65	150	37.2	179	48.2
	66–75	97	56.7		
					
All	40–55	97	10.7	222	12.8
	56–65	210	25.2	224	41.0
	66–75	175	45.3		

**Table 3:** Sensitivity (SENS) and specificity (SPEC) of coronary risk calculators at the high risk threshold (SCORE 5%, 20% remainders) to detect TPA80.

Switzerland (*N* = 2202)	SENS	95%CI	SPEC	95%CI
SCORE	32.64	28.5–37.0	92.08	90.7–93.3
SCORE-HDL	19.21	15.8–23.0	96.33	95.3–97.2
PCE	17.36	14.1–21.0	96.39	95.4–97.2
FRAM-CHD	21.07	17.5–25.0	95.81	94.8–96.7
FRAM-CVD	39.05	34.7–43.6	87.14	85.5–88.7
PROCAM	12.81	10.0–16.1	97.09	96.2–97.8
AGLA	5.17	3.4–7.5	98.95	98.3–99.4

Germany (*N* = 2942)	SENS	95%CI	SPEC	95%CI
SCORE	11.73	8.9–15.1	99.4	99.0–99.7
SCORE-HDL	7.3	5.1–10.1	99.64	99.3–99.8
PCE	6.42	4.3–9.1	93.29	92.2–94.2
FRAM-CHD	18.14	14.7–22.0	98.71	98.2–99.1
FRAM-CVD	39.38	34.8–44.1	94.9	94.0–95.7
PROCAM	15.27	12.1–18.9	98.8	98.3–99.2

## Appendix A Supplementary data

Supplementary tables Image 1
