# Supplementary material for: Extensive carotid atherosclerosis and the diagnostic accuracy of coronary risk calculators
Source: Prev Med Rep. 2017 Mar 14;6:182–6. doi: 10.1016/j.pmedr.2017.03.006 (PMC5367800; doi:10.1016/j.pmedr.2017.03.006)
Supplement: Supplementary file 1 — Supplementary tables [file mmc1.docx]

ONLINE SUPPLEMENT

Supplemental Table I: Area under the curve (AUC) of risk calculators to detect TPA80 and level of significance for the differences in AUC results

| **Switzerland** | **AUC** | **95%CI** | **p** |
| --- | --- | --- | --- |
| **SCORE** | 0.773 | 0.755 to 0.790 | <0.0001 |
| **SCORE-HDL** | 0.782 | 0.764 to 0.799 | <0.0001 |
| **FRAM-CHD** | 0.765 | 0.747 to 0.782 | <0.0001 |
| **FRAM-CVD** | 0.767 | 0.749 to 0.785 | <0.0001 |
| **PCE** | 0.778 | 0.760 to 0.795 | <0.0001 |
| **AGLA** | 0.743 | 0.725 to 0.762 | <0.0001 |

| **Switzerland** | **SCORE** | **SCORE-HDL** | **FRAM-CHD** | **FRAM-CVD** | **PCE** |
| --- | --- | --- | --- | --- | --- |
| **SCORE** |  |  |  |  |  |
| **SCORE-HDL** | P = 0.0075 |  |  |  |  |
| **FRAM-CHD** | P = 0.3312 | P = 0.0062 |  |  |  |
| **FRAM-CVD** | P = 0.4613 | P = 0.0100 | P = 0.0991 |  |  |
| **PCE** | P = 0.3653 | P = 0.3014 | P = 0.0331 | P = 0.0610 |  |
| **AGLA** | P = 0.0030 | P < 0.0001 | P = 0.0035 | P = 0.0008 | P < 0.0001 |

| **Germany** | **AUC** | **95%CI** | **p** |
| --- | --- | --- | --- |
| **SCORE** | 0.868 | 0.856 to 0.880 | <0.0001 |
| **SCORE-HDL** | 0.867 | 0.854 to 0.879 | <0.0001 |
| **FRAM-CHD** | 0.856 | 0.843 to 0.868 | <0.0001 |
| **FRAM-CVD** | 0.859 | 0.846 to 0.871 | <0.0001 |
| **PCE** | 0.769 | 0.754 to 0.784 | <0.0001 |
| **PROCAM** | 0.830 | 0.816 to 0.844 | <0.0001 |

| **Germany** | **SCORE** | **SCORE-HDL** | **FRAM-CHD** | **FRAM-CVD** | **PCE** |
| --- | --- | --- | --- | --- | --- |
| **SCORE** |  |  |  |  |  |
| **SCORE-HDL** | P = 0.6090 |  |  |  |  |
| **FRAM-CHD** | P = 0.0066 | P = 0.0009 |  |  |  |
| **FRAM-CVD** | P = 0.0320 | P = 0.0150 | P = 0.0024 |  |  |
| **PCE** | P < 0.0001 | P < 0.0001 | P < 0.0001 | P < 0.0001 |  |
| **PROCAM** | P < 0.0001 | P < 0.0001 | P < 0.0001 | P < 0.0001 | P 0.0001 |

**Supplemental Table II: PROCAM and SCORE decision thresholds, sensitivities, specificities and positive and negative predictive values to detect TPA80 for Germany**

| **Women** | | **PROCAM** | | | | **Women** | | **SCORE** | | | | |
| --- | --- | --- | --- | --- | --- | --- | --- | --- | --- | --- | --- | --- |
| **decision threshold** |  | **SENS** | **SPEC** | **PPV** | **NPV** | **decision threshold** |  | **SENS** | **SPEC** | **PPV** | **NPV** |  |
| **40 - 55** |  |  |  |  |  | **40 – 55** |  |  |  |  |  |  |
| 3.0 |  | 37 | 96 | 28 | 97 | 0.75 |  | 41 | 95 | 27 | 97 |  |
| 4.0 |  | 26 | 98 | 41 | 97 | 1.00 |  | 15 | 99 | 44 | 96 |  |
| 5.0 |  | 15 | 99 | 33 | 96 | 1.25 |  | 4 | 99 | 25 | 96 |  |
| 7.5 |  | 7 | 99 | 33 | 96 | 1.88 |  | 0 | 100 | 0 | 96 |  |
| 10.0 |  | 4 | 100 | 33 | 96 | 2.50 |  | 0 | 100 | - | 96 |  |
| 15.0 |  | 0 | 100 | 0 | 96 | 3.75 |  | 0 | 100 | - | 96 |  |
| 20.0 |  | 0 | 100 | - | 96 | 5.00 |  | 0 | 100 | - | 96 |  |
| **56 - 65** |  |  |  |  |  | **56 - 65** |  |  |  |  |  |  |
| 3.0 |  | 62 | 53 | 31 | 80 | 0.75 |  | 89 | 17 | 27 | 81 |  |
| 4.0 |  | 42 | 67 | 31 | 77 | 1.00 |  | 71 | 35 | 28 | 78 |  |
| 5.0 |  | 38 | 75 | 34 | 78 | 1.25 |  | 58 | 51 | 29 | 78 |  |
| 7.5 |  | 20 | 91 | 43 | 77 | 1.88 |  | 31 | 78 | 33 | 77 |  |
| 10.0 |  | 20 | 95 | 56 | 77 | 2.50 |  | 13 | 88 | 29 | 75 |  |
| 15.0 |  | 7 | 98 | 50 | 75 | 3.75 |  | 4 | 95 | 25 | 74 |  |
| 20.0 |  | 4 | 98 | 50 | 75 | 5.00 |  | 2 | 99 | 50 | 75 |  |
| **Men** | | **PROCAM** | | | | **Men** | | **SCORE** | | | | |
| **decision threshold** |  | **SENS** | **SPEC** | **PPV** | **NPV** | **decision threshold** |  | **SENS** | **SPEC** | **PPV** | **NPV** |  |
| **40 - 55** |  |  |  |  |  | **40 – 55** |  |  |  |  |  |  |
| 3.0 |  | 84 | 45 | 24 | 93 | 0.75 |  | 88 | 48 | 27 | 95 |  |
| 4.0 |  | 76 | 57 | 27 | 92 | 1.00 |  | 76 | 63 | 30 | 93 |  |
| 5.0 |  | 71 | 68 | 32 | 92 | 1.25 |  | 66 | 74 | 35 | 91 |  |
| 7.5 |  | 52 | 82 | 38 | 89 | 1.88 |  | 43 | 90 | 47 | 88 |  |
| 10.0 |  | 45 | 89 | 48 | 88 | 2.50 |  | 28 | 96 | 61 | 86 |  |
| 15.0 |  | 23 | 96 | 55 | 85 | 3.75 |  | 11 | 100 | 88 | 84 |  |
| 20.0 |  | 14 | 99 | 69 | 84 | 5.00 |  | 4 | 100 | 88 | 83 |  |
| **56 - 65** |  |  |  |  |  | **56 - 65** |  |  |  |  |  |  |
| 3.0 |  | 92 | 11 | 49 | 60 | 0.75 |  | 100 | 0 | 48 | - |  |
| 4.0 |  | 91 | 17 | 50 | 65 | 1.00 |  | 99 | 0 | 48 | 0 |  |
| 5.0 |  | 84 | 27 | 52 | 65 | 1.25 |  | 99 | 2 | 49 | 80 |  |
| 7.5 |  | 68 | 45 | 54 | 60 | 1.88 |  | 91 | 21 | 52 | 71 |  |
| 10.0 |  | 57 | 63 | 59 | 61 | 2.50 |  | 74 | 42 | 54 | 63 |  |
| 15.0 |  | 33 | 82 | 63 | 57 | 3.75 |  | 42 | 78 | 64 | 59 |  |
| 20.0 |  | 21 | 91 | 69 | 55 | 5.00 |  | 24 | 93 | 77 | 57 |  |

**Supplemental Table III: AGLA and SCORE decision thresholds, sensitivities, specificities and positive and negative predictive values to detect TPA80 for Switzerland, women only**

| **Women** | | **AGLA** | | | | **Women** | | **SCORE** | | | | | |
| --- | --- | --- | --- | --- | --- | --- | --- | --- | --- | --- | --- | --- | --- |
| **decision threshold** |  | **SENS** | **SPEC** | **PPV** | **NPV** | **decision threshold** |  | **SENS** | **SPEC** | **PPV** | **NPV** |  |  |
| **40 - 55** |  |  |  |  |  | **40 – 55** |  |  |  |  |  | |  |
| 3.0 |  | 22 | 95 | 18 | 96 | 0.75 |  | 39 | 89 | 15 | 97 | |  |
| 4.0 |  | 17 | 96 | 17 | 96 | 1.00 |  | 28 | 95 | 20 | 97 | |  |
| 5.0 |  | 0 | 98 | 0 | 95 | 1.25 |  | 22 | 99 | 44 | 96 | |  |
| 7.5 |  | 0 | 99 | 0 | 95 | 1.88 |  | 6 | 99 | 33 | 96 | |  |
| 10.0 |  | 0 | 99 | 0 | 95 | 2.50 |  | 6 | 100 | 50 | 96 | |  |
| 15.0 |  | 0 | 100 | - | 96 | 3.75 |  | 0 | 100 | - | 96 | |  |
| 20.0 |  | 0 | 100 | - | 96 | 5.00 |  | 0 | 100 | - | 96 | |  |
| **56 - 65** |  |  |  |  |  | **56 - 65** |  |  |  |  |  | |  |
| 3.0 |  | 53 | 74 | 25 | 91 | 0.75 |  | 97 | 14 | 15 | 96 | |  |
| 4.0 |  | 45 | 84 | 31 | 90 | 1.00 |  | 90 | 26 | 17 | 94 | |  |
| 5.0 |  | 37 | 89 | 35 | 90 | 1.25 |  | 85 | 41 | 19 | 94 | |  |
| 7.5 |  | 20 | 94 | 34 | 88 | 1.88 |  | 57 | 68 | 23 | 91 | |  |
| 10.0 |  | 10 | 97 | 38 | 87 | 2.50 |  | 32 | 85 | 26 | 89 | |  |
| 15.0 |  | 2 | 99 | 33 | 86 | 3.75 |  | 15 | 97 | 43 | 88 | |  |
| 20.0 |  | 0 | 99 | 0 | 86 | 5.00 |  | 3 | 98 | 20 | 86 | |  |
| **66 - 75** |  |  |  |  |  | **66 - 75** |  |  |  |  |  | |  |
| 3.0 |  | 74 | 39 | 41 | 73 | 0.75 |  | 100 | 0 | 36 | - | |  |
| 4.0 |  | 63 | 48 | 41 | 69 | 1.00 |  | 100 | 0 | 36 | - | |  |
| 5.0 |  | 51 | 64 | 44 | 70 | 1.25 |  | 100 | 0 | 36 | - | |  |
| 7.5 |  | 32 | 81 | 49 | 68 | 1.88 |  | 96 | 3 | 36 | 57 | |  |
| 10.0 |  | 13 | 88 | 37 | 64 | 2.50 |  | 91 | 16 | 38 | 76 | |  |
| 15.0 |  | 9 | 98 | 70 | 65 | 3.75 |  | 68 | 55 | 46 | 75 | |  |
| 20.0 |  | 5 | 99 | 67 | 65 | 5.00 |  | 47 | 74 | 51 | 71 | |  |

**Supplemental Table IV: AGLA and SCORE decision thresholds, sensitivities, specificities and positive and negative predictive values to detect TPA80 for Switzerland, men only**

| **Men** | | **AGLA** | | | | **Men** | | **SCORE** | | | |
| --- | --- | --- | --- | --- | --- | --- | --- | --- | --- | --- | --- |
| **decision threshold** |  | **SENS** | **SPEC** | **PPV** | **NPV** | **decision threshold** |  | **SENS** | **SPEC** | **PPV** | **NPV** |
| **40 - 55** |  |  |  |  |  | **40 – 55** |  |  |  |  |  |
| 3.0 |  | 53 | 74 | 25 | 91 | 0.75 |  | 87 | 31 | 19 | 93 |
| 4.0 |  | 45 | 84 | 31 | 90 | 1.00 |  | 68 | 47 | 19 | 89 |
| 5.0 |  | 37 | 89 | 35 | 90 | 1.25 |  | 54 | 60 | 20 | 88 |
| 7.5 |  | 20 | 94 | 34 | 88 | 1.88 |  | 34 | 79 | 24 | 87 |
| 10.0 |  | 10 | 97 | 38 | 87 | 2.50 |  | 24 | 90 | 30 | 86 |
| 15.0 |  | 2 | 99 | 33 | 86 | 3.75 |  | 10 | 97 | 40 | 85 |
| 20.0 |  | 0 | 99 | 0 | 86 | 5.00 |  | 3 | 100 | 67 | 85 |
| **56 - 65** |  |  |  |  |  | **56 - 65** |  |  |  |  |  |
| 3.0 |  | 74 | 39 | 41 | 73 | 0.75 |  | 100 | 0 | 37 | - |
| 4.0 |  | 63 | 48 | 41 | 69 | 1.00 |  | 99 | 0 | 37 | 33 |
| 5.0 |  | 51 | 64 | 44 | 70 | 1.25 |  | 99 | 2 | 37 | 67 |
| 7.5 |  | 32 | 81 | 49 | 68 | 1.88 |  | 90 | 13 | 38 | 68 |
| 10.0 |  | 13 | 88 | 37 | 64 | 2.50 |  | 76 | 34 | 41 | 71 |
| 15.0 |  | 9 | 98 | 70 | 65 | 3.75 |  | 45 | 69 | 46 | 68 |
| 20.0 |  | 5 | 99 | 67 | 65 | 5.00 |  | 25 | 84 | 49 | 66 |
| **66 - 75** |  |  |  |  |  | **66 - 75** |  |  |  |  |  |
| 3.0 |  | 71 | 55 | 23 | 91 | 0.75 |  | 100 | 0 | 57 | - |
| 4.0 |  | 56 | 65 | 23 | 89 | 1.00 |  | 100 | 0 | 57 | - |
| 5.0 |  | 44 | 71 | 22 | 87 | 1.25 |  | 100 | 0 | 57 | - |
| 7.5 |  | 28 | 83 | 24 | 86 | 1.88 |  | 100 | 0 | 57 | - |
| 10.0 |  | 23 | 91 | 32 | 86 | 2.50 |  | 99 | 1 | 57 | 50 |
| 15.0 |  | 6 | 96 | 25 | 85 | 3.75 |  | 96 | 5 | 57 | 50 |
| 20.0 |  | 3 | 99 | 29 | 84 | 5.00 |  | 81 | 30 | 60 | 55 |

**Supplemental Table V: Sensitivity of various thresholds of coronary risk calculators by age group 40-55, by country (CH and DE) to detect TPA80 at a prevalence of CH 11% and DE 13%**

| 40 - 55 |  |  |  |  |  |  |  |  |  |  |  |  |
| --- | --- | --- | --- | --- | --- | --- | --- | --- | --- | --- | --- | --- |
| Other / SCORE | CH-FRAM-CHD | CH-FRAM-CVD | CH-SCORE | CH-SCORE-HDL | CH-PCE | CH-AGLA | DE-FRAM-CHD | DE-FRAM-CVD | DE-SCORE | DE-SCORE-HDL | DE-PCE | DE-PROCAM |
| 0 - 2.5/1% | 96 | 100 | 61 | 55 | 90 | 64 | 98 | 100 | 69 | 59 | 92 | 81 |
| 0 - 5.0/2% | 86 | 95 | 26 | 21 | 61 | 36 | 91 | 96 | 34 | 27 | 68 | 64 |
| 0 - 7.5/3% | 66 | 81 | 11 | 8 | 37 | 23 | 68 | 88 | 17 | 13 | 48 | 47 |
| 0 - 10.0/4% | 48 | 70 | 7 | 4 | 24 | 19 | 51 | 75 | 6 | 4 | 34 | 40 |
| 0 - 12.5/5% | 28 | 55 | 2 | 1 | 14 | 8 | 39 | 59 | 3 | 2 | 22 | 27 |
| 0 - 15.0/6% | 19 | 40 | 1 | 1 | 8 | 5 | 28 | 50 | 2 | 1 | 14 | 20 |
| 0 - 17.5/7% | 13 | 28 | 1 | 1 | 3 | 3 | 22 | 39 | 1 | 0 | 8 | 15 |
| 0 - 20.0/8% | 10 | 22 | 1 | 0 | 3 | 2 | 13 | 30 | 1 | 0 | 4 | 12 |
| 0 - 22.5/9% | 3 | 18 | 0 | 0 | 1 | 1 | 9 | 25 | 0 | 0 | 2 | 10 |
| 0 - 25.0/10% | 3 | 13 | 0 | 0 | 1 | 1 | 5 | 21 | 0 | 0 | 1 | 7 |
| 0 - 27.5/11% | 2 | 10 | 0 | 0 | 0 | 0 | 4 | 13 | 0 | 0 | 1 | 6 |
| 0 - 30.0/12% | 0 | 8 | 0 | 0 | 0 | 0 | 1 | 9 | 0 | 0 | 1 | 4 |

Other denotes FRAM, PROCAM, AGLA, PCE

**Supplemental Table VI: Sensitivity of various thresholds of coronary risk calculators by age group 56-65, by country (CH and DE) to detect TPA80 at a prevalence of CH 25% and DE 41%**

| 56 - 65 |  |  |  |  |  |  |  |  |  |  |  |  |
| --- | --- | --- | --- | --- | --- | --- | --- | --- | --- | --- | --- | --- |
| Other / SCORE | CH-FRAM-CHD | CH-FRAM-CVD | CH-SCORE | CH-SCORE-HDL | CH-PCE | CH-AGLA | DE-FRAM-CHD | DE-FRAM-CVD | DE-SCORE | DE-SCORE-HDL | DE-PCE | DE-PROCAM |
| 0 - 2.5/1% | 100 | 100 | 96 | 90 | 100 | 77 | 100 | 100 | 94 | 86 | 96 | 89 |
| 0 - 5.0/2% | 91 | 99 | 77 | 62 | 88 | 51 | 92 | 99 | 74 | 62 | 88 | 75 |
| 0 - 7.5/3% | 80 | 92 | 50 | 34 | 72 | 36 | 83 | 93 | 51 | 34 | 76 | 58 |
| 0 - 10.0/4% | 65 | 87 | 31 | 21 | 51 | 23 | 72 | 86 | 31 | 22 | 59 | 50 |
| 0 - 12.5/5% | 48 | 76 | 19 | 12 | 35 | 16 | 59 | 79 | 20 | 12 | 38 | 40 |
| 0 - 15.0/6% | 34 | 63 | 12 | 8 | 22 | 10 | 45 | 70 | 13 | 7 | 23 | 28 |
| 0 - 17.5/7% | 23 | 50 | 6 | 3 | 14 | 7 | 32 | 60 | 8 | 4 | 13 | 21 |
| 0 - 20.0/8% | 16 | 39 | 5 | 2 | 6 | 4 | 23 | 48 | 4 | 2 | 8 | 18 |
| 0 - 22.5/9% | 11 | 30 | 4 | 1 | 3 | 4 | 16 | 42 | 3 | 1 | 4 | 13 |
| 0 - 25.0/10% | 10 | 22 | 2 | 1 | 1 | 2 | 11 | 31 | 2 | 1 | 2 | 9 |
| 0 - 27.5/11% | 6 | 17 | 2 | 0 | 1 | 1 | 8 | 25 | 2 | 1 | 1 | 5 |
| 0 - 30.0/12% | 4 | 14 | 0 | 0 | 0 | 1 | 5 | 18 | 1 | 0 | 0 | 4 |

Other denotes FRAM, PROCAM, AGLA, PCE
